# Histological and radiographic assessment of the regenerative potential of sodium hexametaphosphate (SHMP) as a novel direct pulp capping material in an animal model

**DOI:** 10.1186/s12903-024-05297-0

**Published:** 2025-01-03

**Authors:** Mostafa Kamel Mohamed, Mohamed Abdelfattah Abdelrahman, Abdel-Razik Hashem Abdel-Razik, Ahmad Abdel Hamid Elheeny

**Affiliations:** 1 Paediatric and Community Dentistry, Faculty of Dentistry, Minia University, Ard Shalaby, El Minia, 61519 Egypt; 2 Surgery, Anestheiology, and Radiology Department, Faculty of Veterinary Medicine, Minia University, El Minya, Egypt; 3 Faculty of Veterinary Medicine, Beni-Suef University, Beni-Suef, Egypt; 4 Paediatric and Community Dentistry, Faculty of Dentistry, Sphinx University, Asyut, Egypt

**Keywords:** Mineral trioxide aggregate, Pulp capping, Regenerative endodontics

## Abstract

**Background:**

This study aimed to assess the histological and radiographic effects of sodium hexametaphosphate (SHMP) as a direct pulp capping (DPC) agent in immature permanent dog premolars.

**Methods:**

A split-mouth design was employed with three healthy 4-month-old Mongrel dogs, each having 36 premolars. The premolars were randomly assigned to either SHMP or MTA. The specimens were stained with hematoxylin and eosin (H&E) and Masson’s trichrome, and histologically examined three months after the animals were sacrificed. To assess root maturity, radiographic changes in root length (RL), root surface area (RSA), and apical foramen width (AFW) were measured at baseline and after 3 months. Quantitative data were analyzed using the paired-sample t-test, while the qualitative data based on Stanley's histological scoring system were tested using the Monte Carlo exact test. The level of significance was set at 5%.

**Results:**

Histological findings showed no significant differences between the two groups, except for the average thickness of the predentin and odontoblastic layers, which was significantly higher in the SHMP specimens (*P* < 0.0001). The frequencies of fully calcified dentin bridges and regularly arranged dentinal tubules were significantly higher in the SHMP specimens (*P* < 0.05). Both materials showed comparable radiographic measurements (*P* > 0.05), except for the change in RL, which was significantly longer in the SHMP group (*P* < 0.05).

**Conclusions:**

There were no significant differences between SHMP and MTA in some respects. Histological evaluation showed that SHMP provided better bioinductive and biocompatible properties compared to MTA. Radiographically, both materials showed comparable root maturogenesis outcomes, except for the significant increase in RL in the SHMP group. SHMP may be a suitable alternative material for DPC in the treatment of immature permanent teeth.

## Introduction

Direct pulp capping (DPC) is one of the most conservative vital pulp therapy (VPT) techniques aims at protecting the pulp tissue in healthy status in terms of maintaining the integrity of the vascular and neural supply in the dentin-pulp complex as well as supporting the immune defence mechanism of the dental pulp [1, 2]. DPC involves the application of a biocompatible dressing material over an injured pulp to prompt the regenerative power of human dental pulp cells (HDPCs) required for the healing process [1, 3]. The standards of optimal DPC material include biocompatibility, preventing microleakage, positive antimicrobial activity, and the bioinductive power of a calcified barrier with highly qualified properties [4, 5].

Several factors are affecting the decision-making of DPC procedures. According to the recent data gathered from a multinational survey from sixteen countries [6], pulp exposure resulting from carious lesions is the most significant determinant in clinical decisions regarding DPC. Other prognostic factors included the location of exposure, with dental practitioners showing a higher tendency to perform DPC for occlusally located caries compared caries located on the axial surfaces.

Mineral trioxide aggregate (MTA) is still the benchmark capping agent because of its excellent physiochemical characteristics in terms of its effective potential for dentinogensis and its ability to cause less pulpal inflammation [7, 8]. Additionally, the high success rate results of several clinical trials boost the reliability of MTA as a capping material in VPT, especially DPC [8]. However, there are some negative issues regarding the use of MTA. These drawbacks include prolonged duration required for setting, inadequate adhesion to the dentin, inconsistent mix and handling difficulties, the possibility of crown discoloration, toxicity due to bismuth leaching that has a negative impact on the cell viability, and the concerns that have been raised about the potential leakage of arsenic, its little bioinductive properties, and the high cost [9–11]. One of the major disadvantages of hydraulic calcium silicate cement such as MTA is the color change, especially in the medium and long term in contact with blood, because due to its radio-opacifier bismuth oxide, which is oxidized or reduced upon exposure to oxidizing agents including dentin collagen and sodium hypochlorite [12]. Minimizing the diffusion of bismuth ions into the dentin tooth structure through dentinal tubule blockage using dentin bonding agents and applying a layer of glass ionomer linear can be effective in reducing coronal discoloration [13]. Other strategies to reduce the crown discoloration include modifying the composition of gray MTA by eliminating metallic oxide components producing white MTA. However, tooth crowns still suffer from some degree of discoloration [14]. Color alteration becomes worse in the presence of blood due to the higher blood absorption and subsequent hemolysis of MTA because it remains porous for a longer period [12]. Therefore, MTA should be placed 2−3 mm below the cementoenamel junction [12, 15]. Another approach is modifying the powder of white MTA by the addition of zinc oxide or aluminum fluoride [16, 17]. The introduction of new hydraulic calcium silicate cement in which bismuth oxide is replaced with other radiopacifiers such as zirconium oxide in Biodentine [18] and tantalum peroxide in bioaggregate [19].

Another problem associated with MTA is the need for delayed setting time (2 h and 45 min) within a hydrated media. Therefore, the need for an extra appointment is required for final restoration placement [20]. New calcium silicate-based cements, such as Biodentine, which permits immediate bonding procedures [20].

Accordingly, searching for capping material with higher qualities is still mandatory and represents a challenge for researchers. Sodium hexametaphosphate (SHMP) is a candidate of inorganic polyphosphates (poly[P]) [21]. Poly(P) are linear polymers that are located in various body cells and consist of orthophosphate (Pi) residues connected with highly energetic bonds [21]. Poly(P) has a significant role in the differentiation and maturation of osteoblasts, reflecting its osteoinductive potential and osseous calcification and prompting differentiation of human gingival fibroblasts [21, 22]. The odontogenic power of poly(P) compounds such as SHMP to induce proliferation and differentiation of odontoblast-like cells was postulated in two previous in vitro studies [21, 23]. The first tested the effect of two poly(P) candidates, SHMP and sodium triphosphate (STP), on the regenerative potential of HDPCs [21]. The increase in cell count, alkaline phosphatase (ALP) activity, differentiation markers such as osteonectin, osteopontin (OPN), and osteocalcin (OCN), and the angiogenic factors in HDPCs indicated the capability of SHMP and STP in inducing HDPC proliferation and differentiation into odontoblast-like cells. The other study tested the role of matrix metalloproteinase (MMP)−3 on the growth and maturation of odontoblast-like cells [23]. It was found that poly(P) induces odontoblastic biomarkers such as ALP, dentin sialophosphoprotein (DSPP), and dentin matrix protein-1 (DMP-1) mRNA that induce precipitation of osseous deposits. Accordingly, SHMP can be used in regenerative endodontic procedures.

Until now, no in vivo study has been conducted to evaluate the biocompatibility and bioinductive potential of poly(P) on exposed dental pulp tissues. Therefore, the current study aimed to evaluate the histological and radiographic influence of SHMP as DPC agents in young permanent dogs' premolars. The null hypotheses (*H*_*0*_) of the current study suggested no histological (primary outcome) or radiographic differences (secondary outcome), respectively, between SHMP and MTA as capping materials in DPC immature dogs' teeth.

## Materials and methods

### Animal model

**Fig. 1:**
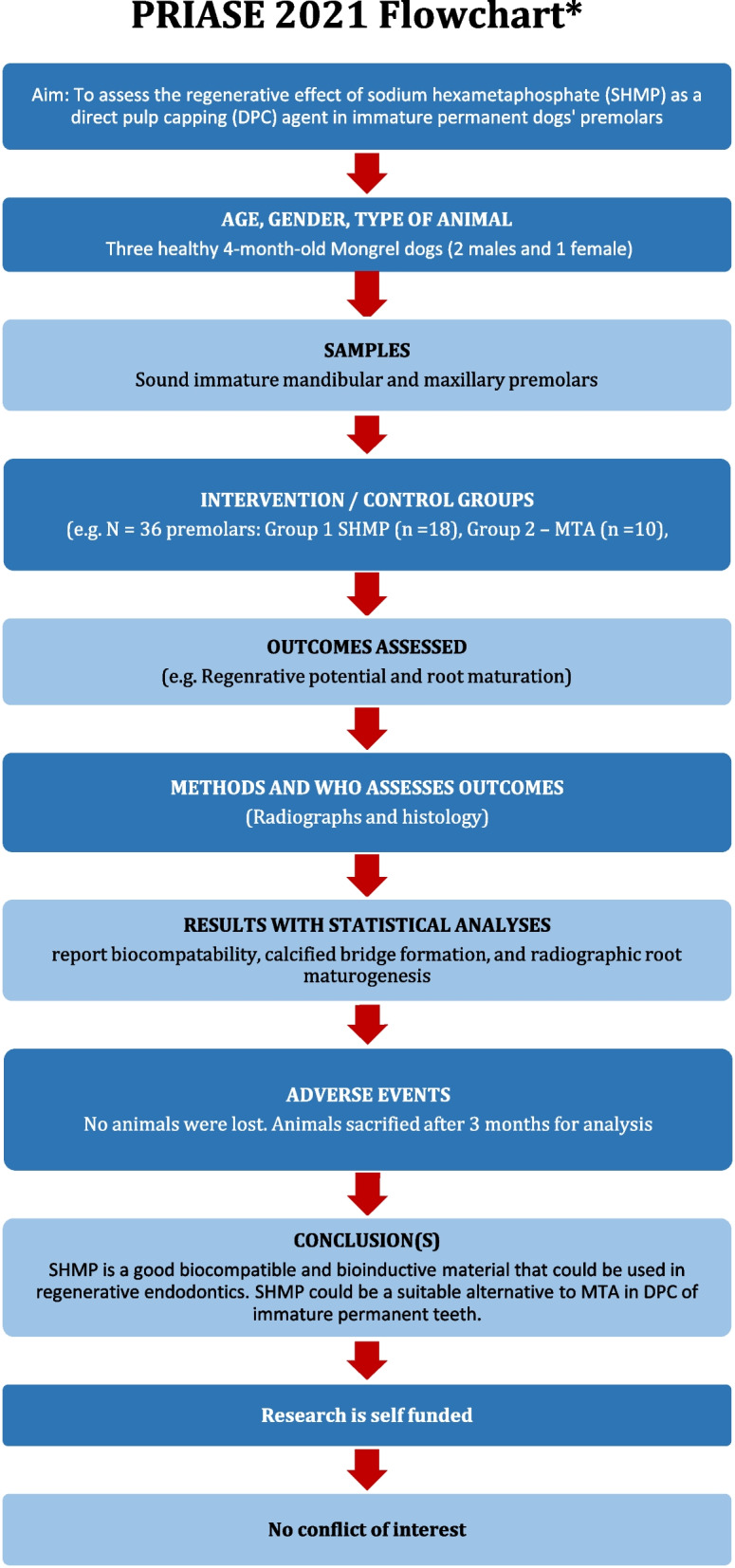
PRIASE flowchart of the study

The work has been reported in line with the ARRIVE guidelines 2.0. A summary of the study steps according to the PRIASE flowchart was illustrated as Fig. 1.

Animals were housed in standard individual cages with an adequate daily amount of food and water ad libitum. Parasiticide of 1 mL/50 kg of Ivermectin (Ivomec supra® 1% injection, Merial, USA) administered subcutaneously. On the day of operation and after weighting each dog, an intravenous cannula (20 gauges) was placed in the recurrent tarsal (saphenous) vein.

### Study design, sampling and randomization

A split-mouth study design included a total of 36 premolars of three healthy 4-month-old Mongrel dogs (two males and one female) weighing approximately 7−9 kg. The number of teeth was calculated using a G*Power 3.1.9.4 after considering a medium effect size of the dependent means of the induced calcified bridge thickness after capping with the two materials. At a 5% alpha level of significance and a power of 80%, a total of 36 teeth were required. Teeth were assigned to either the right or left side of the jaw and were randomly treated with SHMP (intervention group, included 18 teeth) or MTA (control group, included 18 teeth) using a simple randomization approach. DPC was performed via traumatic exposure of sound immature maxillary and mandibular premolars of dogs (expect the fourth maxillary and mandibular premolars) with no apparent coronal or radicular developmental anomalies that have been confirmed with periapical radiographs.

### Surgical procedures

Food and water were held for 12 and 6 h before induction of anesthesia. Each dog was weighed to calculate the dosage of the drug, then a butterfly cannula (winged infusion set gauge 20) was inserted into the recurrent tarsal (saphenous) vein. Dogs were then premedicated by a slow intravenous administration of 1 mg/kg of xylazine HCl (Xyla-ject; ADWIA Co. New Cairo, Egypt) diluted in 3 ml saline. Ten minutes later, anaesthesia was induced by intravenous 10 mg/kg Ketamin HCl (Ketamine®; Hamelin, UK) and maintained by 24 mg/kg propofol (Diprivan® 10 mg/ml, Aspen Pharma, South Africa) at a dose of at a constant rate of infusion using an infusion pump. The dose of propofol was calculated and diluted in a net volume of 200 mg of sterile normal sterile saline and was infused at a rate of 100 mL per hour.

### Postoperative care

Cefotaxime sodium (Cefotax® 500 mg vial, Abbott, USA) antibiotic was injected intramuscularly (IM) once daily for three days with a dosage rate of 50 mg/kg. Intramuscular injection of 7.5 mg of meloxicam (Mobitil® 15 mg ampule, Medical Union Pharmaceuticals, Ismailia, Egypt) were given twice a day as a non-steroidal anti-inflammatory and analgesic medication. Additionally, for three days, Povidine Iodine mouthwash and gargle (Betadine® Mundipharma, Dublin, Ireland) was administered twice a day as an oral antibacterial. Operated animals were followed up during and after the recovery period of anesthesia by well-trained animal caretakers at the veterinary medicine clinic of the local institution. The animals were examined on a regular basis to make sure they had a sufficient and healthy appetite and that all clinical parameters and vital signs (heart rate, pulse, temperature of the rectal area, breathing rate, urine, and excrement) were within normal ranges.

### Method of euthanasia

After three months, a butterfly cannula (winged infusion set, gauge 20 gauge) was insteted into the recurrent tarsal (saphenous) vein for each dog, then they were euthanized by I.V. injection of xylazine at a dose of 1 mg/kg (Xyla-ject® 50 ml solution, ADWIA Co., new Cairo, Egypt). After that the dogs became less conscious, one-shot I.V. injection of thiopental sodium was injected (10 mg/kg; 25% solution) (thiopental-sodium® 500 mg vial, Epico, Egypt) which introduced the animals into deep irreversible anesthesia that ended by their death.

Considering a split mouth design, each dog's maxillary and mandibular jaw halves were randomly treated with SHMP or MTA (18 premolars per group).

### Pulp capping procedures

Before starting the clinical procedures, all teeth were radiographically checked for maturity or any root abnormalities. After anesthesia and tooth isolation, a class V cavity was accessed from the buccal surface about 2 mm above the free gingival margin and parallel to the cementoenamel junction using a tungsten carbide pear-shaped bur, ISO #330 L. The access width was approximately 2−3 mm, and when the reddish pulp shadow was visible, a round carbide bur ISO # 1 (0.8 mm in diameter) was used to standardize the size of the pulp exposure to 0.8−1 mm. All cavity preparation procedures were performed at ultra-high speed with a copious water spray. To maximize cutting efficiency and preserve a sterile environment, each bur was used only once for each cavity [24]. After rinsing the cavity with sterile saline and controlling the bleeding with a small cotton pellet under gentle pressure, the capping material was applied. In the experimental group, the crystalline SHMP (Oxford LAB FINE CHEM LLP, India, HSN Code: 28352940 and CAS number: 10124–56-8) was ground into fine powder. The powder was sterilized in a hot air oven before use. The powder with saline was mixed with saline in a 2 (powder):1 (liquid) proportion until a thick, workable mixture was obtained. The mix was applied using the MTA plug and condensed against the exposure site with a small, serrated amalgam condenser size¼ (HENRY SCHEIN®, USA) covered with a Teflon.

In the control group, MTA (white MTA Angelus, Londrina, PR, Brazil) was prepared according to the manufacturer's guidelines and placed over the exposed pulp by an MTA carrier (Angelus, Londrina, Brazil). A moistened cotton pellet was placed over the MTA to permit its setting under the final restorative material. Finally, the access cavities of all teeth were sealed with a glass ionomer restoration (RIVA self-cure, SDI Ltd. Victoria, Australia).

To assess the changes in root maturogenesis, 2D-digital periapical radiographs of the capped teeth were taken preoperatively and at the time of animal scarification. For standardization, all periapical images were taken using the paralleling technique (Vista Scan Mini Easy X-ray System, Bietigheim-Bissingen Germany). A PSP plate #2 (size of 3×4 cm) with a 100% active surface area was attached to a film holder that was mounted to a custom-made silicon-based index.

### Histological analysis and grading

For specimen preparation and histological analysis, dogs were euthanized by thiopental overdose at the predetermined interval. Surgical dissection was used to split the mandibular and maxillary jaws into halves along their midline. The specimens were decalcified in 10% EDTA for 3 months after being fixed for 7 days in 10% neutral buffered formalin. The specimens were dehydrated using ascending grades of ethyl alcohol, cleared in xylene, impregnated in soft paraffin, and finally embedded in hard paraffin.

The capped teeth were extracted and washed thoroughly under running tap water for 3−4 h. To obtain serial sections of 5 μm thickness, extracted teeth were embedded in paraffin and cut buccolingually parallel to their vertical axis through the accessed cavities. Tissue sections were stained with haematoxylin and eosin (H & E) and Masson’s trichrome. The later was used to stain bone and collagen fibers and distiguish cells from the surrounding connective tissue [25]. Representative photomicrographs were taken using a digital camera (LEICA, DFC290 HD system digital camera, Heerbrugg, Switzerland) connected to the light microscope using 4, 10, 20, and 40 objective lenses.

Based on the criteria of the modified scoring system of Stanley [26] (Table 1), the calcified bridge and inflammatory response of the pulp were scored. The mean thickness scores of the calcified bridge, predentin, and odontoblastic layer were measured using ImageJ software (version 1.50i; National Institutes of Health, Bethesda, MD, USA).

**Table 1:** Distribution of histological criteria according to the modified scoring system of Stanley

Histological parameters and scoring	SHMP	MTA	P*
**Dentin bridge (DB) formation**			
Absent (score 0)	0(0)	0(0)	** < 0.05**
< 25% of DB formed (score 1)	0(0)	0(0)	
26–50% of DB formed (score 2)	0(0)	0(0)	
51–75% of DB formed (score 3)	0(0)	6(33.3)	
76–100% of DB formed (score 4)	18(100)	12(66.7)	
**Location of calcified bridge**			
At the interface of exposure pulp (score 1)	18(100)	18(100)	1.00
Not at the interface of exposure pulp (score 2)	0(0)	0(0)	
Both (score 3)	0(0)	0(0)	
**Quality of dentin formation in the bridge**			
Absence of dentinal tubules (score 0)	0(0)	0(0)	** < 0.05**
Dentinal tubules have regular pattern (score 1)	14(77.8)	8(44.4)	
Dentinal tubules have irregular pattern (score 2)	4(22.2)	10(55.6)	
**Dentin chips**			
Absent (score 0)	9(50)	13(72.2)	> 0.05
Present (score 1)	9(50)	5(22.8)	
**Connective tissue in the bridge**			
Absent (score 0)	14(77.8)	9(50)	> 0.05
< 25% (score 1)	4(22.2)	9(50)	
26–50% (score 2)	0(0)	0(0)	
51–75% (score 3)	0(0)	0(0)	
76–100% (score 4)	0(0)	0(0)	
**Pulpal inflammation**			
Absent (score 0)	18(100)	16(88.8)	> 0.05
Mild (score 1)	0(0)	2(11.1)	
Moderate (score 2)	0(0)	0(0)	
Severe (score 3)	0(0)	0(0)	
Abscess formation (score 4)	0(0)	0(0)	
Tissue necrosis (score 5)	0(0)	0(0)	
**Pulp tissue reaction to the material**			
No inflammatory cell infiltration (score 0)	17(94.4)	14(77.8)	> 0.05
Mild inflammatory cell infiltration (score 1)	1(5.6)	4(22.2)	
Moderate inflammatory cell infiltration (score 2)	0(0)	0(0)	
Severe inflammatory cell infiltration (score 3)	0(0)	0(0)	

*SHMP* Sodium hexametaphosphate, *MTA* Mineral trioxide aggregate

^*^Monte Carlo exact test

### Radiographic measures and analysis

**Fig. 2:**
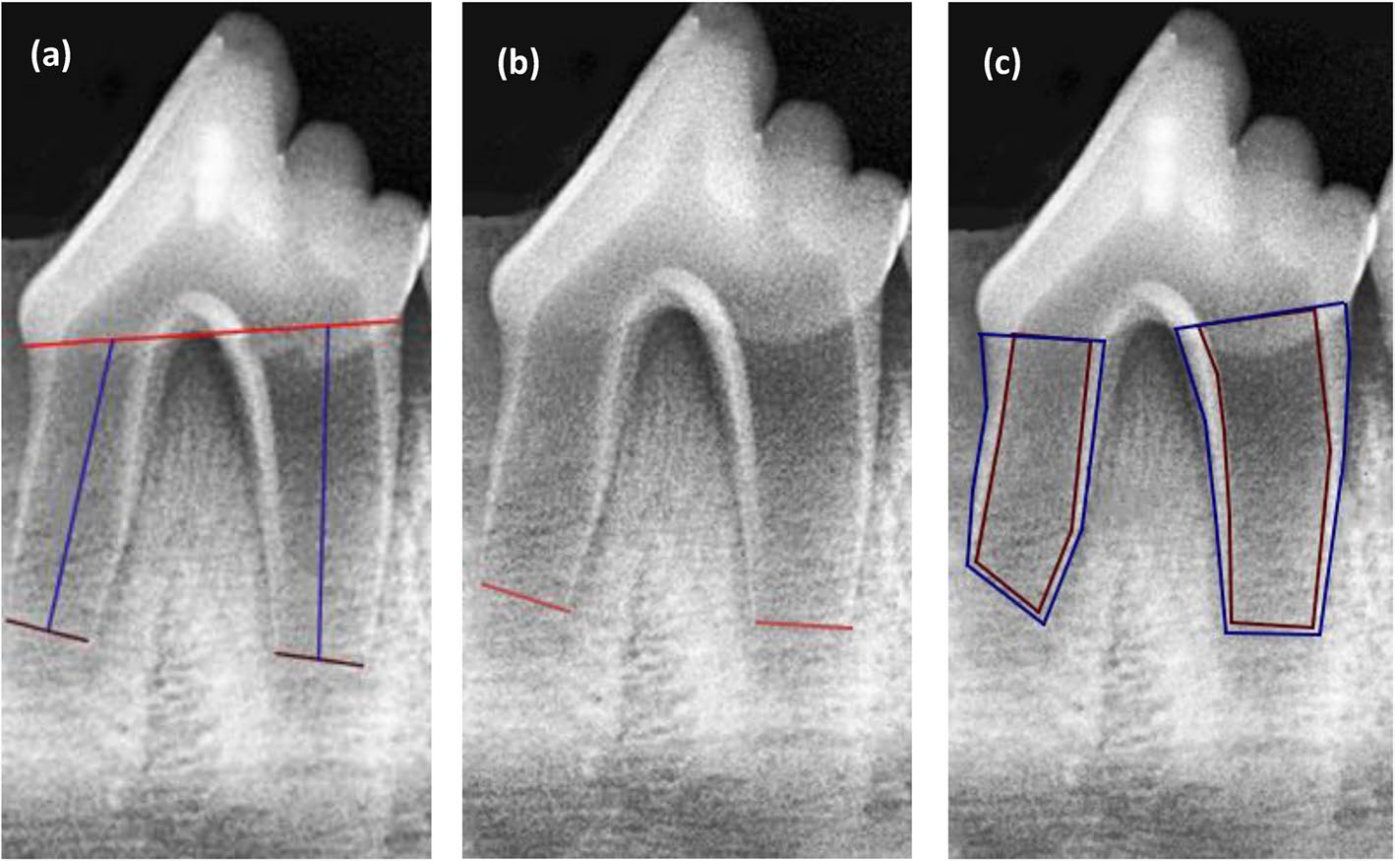
Radiographic measurements reflect root maturation: (**a**) root length (RL) line extended from the CEJ to the apical foramen; (**b**) apical foramen width (AFW) line extended between the mesial and distal root terminals; (**c**) radiographic root area (RRA) calculated by subtracting the total root area from the root canal space

Based on ImageJ software (version 1.50i; National Institutes of Health, Bethesda, MD, USA), the radiographic measurements included the following parameters [27, 28]: Root length (RL): a straight line from the CEJ to the apical foramen (Fig. 2a), apical foramen width (AFW) (Fig. 2b): a line extended between the mesial and distal root ends, and Root surface area (RSA): total root area minus the root canal space (Fig. 2c). Radiographic measures were estimated by an independent expert who was blinded to the used capping materials.

The histological specimens were examined independently by two experienced experts. The degree of agreement was checked using Cohen's Kappa (κ).

### Statistical analysis

Statistical Program for Social Sciences for Windows (SPSS), version 22 (IBM© Corporation, NY, USA) was considered. The measurements of radiographic (RL, RSA, and AFW) and histological data (Thickness of the calcified bridge, predentin, and odontoblastic layer) were tested for normality and variance homogeneity using Kolmogorov–Smirnov and Shapiro–Wilk tests. For histological and radiographic normally distributed quantitative measures obtained, the paired-sample t-test was considered. While the qualitative data of Stanley's scoring system criteria were analysed using the Monte Carlo exact test. The alpha level of significance was set at 5-percent (*P* ≤ 0.05) and a 95% confidence interval (*CI*).

## Results

### Findings of histological analysis

**Fig. 3:**
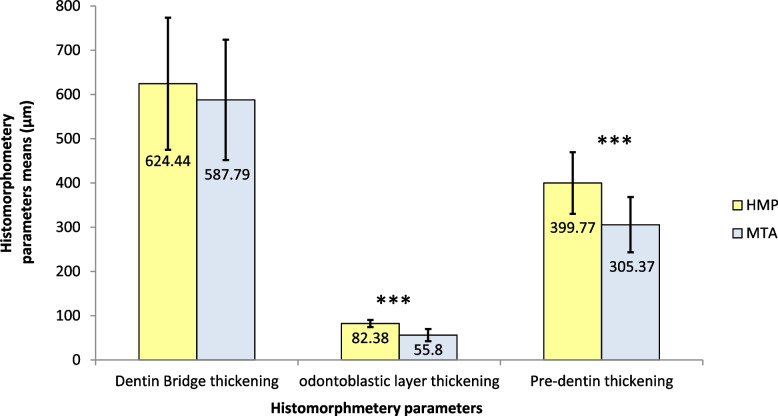
Mean thichness of induced dentin bridges, predentin, and odontablastic layrs formed in resonse to sodium hexametaphosphate (SHMP) and mineral trioxide aggregate (MTA)

The inter-examiner agreement of the histological records was 0.89. Although, the average thickness of the calcific barrier in SHMP specimens (624.44 ± 12.44 µm) was higher than that found in the MTA specimens (587.79 ± 11.34 µm), the difference between the two groups was non-significant (*P* > 0.05). On the other hand, the average thickness values of the predentin and odontoblastic layers were significantly higher in the SHMP specimens compared to the average thickness values in the MTA specimens (*P* < 0.0001) (Fig. 3).

**Fig. 4:**
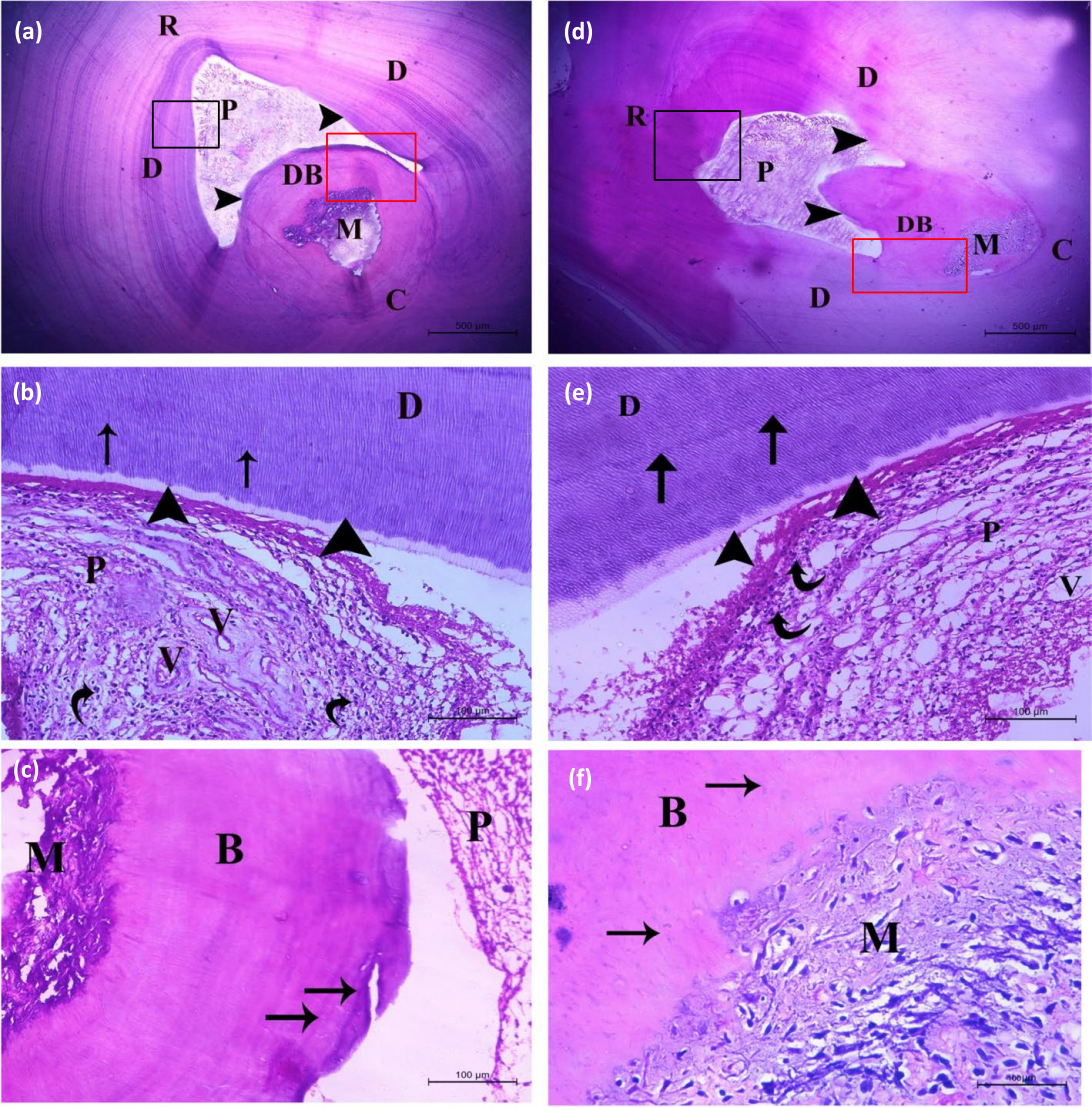
Photomicrograph of the dog's premolars capped with sodium hexametaphosphate (SHMP) (**a**, **b**, and **c**) and mineral trioxide aggregate (MTA) (**d**, **e**, and **f**), stained with H & E. Both materials show that the dentin (D) and pulp (P) are lined with normal odotoblastic layer (arrowheads). The dentin bridge (DB) at the interface between the material (M) and dental pulp (P) is highly organized in the SHMP group (**a**) and less organized in the MTA group (**d**). The magnified black boxed area shows that the two capping materials have loose connective tissue with many normal blood vessels (V) with few chronic inflammatory cells (curved arrow) in the SHMP group (**b**) and many chronic inflammatory cells (curved arrow) in the MTA group (**e**). The magnified red boxed area shows that the calcified bridge (B) induced by the two capping materials are formed from dentin chips. In the SHMP group, the dentin bridge is completely calcified with regularly arranged dentinal tubules and continuous odontoblastic cell layer (Black arrows) (**c**). In contrast, the dentin bridge in MTA group is incompletely calcified and the arrangement of dentinal tubules is less regularly arranged with no demarcating line between the dentin bridge and pulp (Black arrows) (**f**) (Scale bars: (**a**) and (**d**) 500 μm; (**b**), (**c**), (**e**), and (**f**) 100 100 μm)

At the interface between the exposed pulps and both capping materials, the dentin bridge was fully calcified in 100% (18/18) of SHMP specimens, compared to 66.7% (12/18) of MTA specimens. Six MTA specimens (33.3%) showed partial formation of the dentin barrier. The difference between the two groups was statistically significant (*P* < 0.05). The calcified barriers showed deeper H & E staining of in the SHMP group with more homogenous and regular patten of the dentinal tubules compared to the MTA group. Compared to MTA, the dentin bridge induced by SHMP showed a more palisading arrangement of the odontoblastic layer at the interface between dentin and pulp. There were numerous dentinal tubules within the developed hard tissue with homogenous well-arranged pattern similar to the dentin tubular structure (Table 1) and (Fig. 4). The frequency of regularly arranged dentinal tubules in SHMP specimens [(*n* = 14 (77.8%)] was significantly higher compared to those shown in the MTA specimens [*n* = 8 (44.4%)] (*P* < 0.05).

Only two MTA specimens (11.1%) showed mild pulpal inflammation, while all SHMP specimens (100%) were free from pulpal inflammation (*P* > 0.05). No inflammatory cell infiltration was observed in 94.4% and 77.8% of dental pulps in SHMP and MTA specimens, respectively (*P* > 0.05).

**Fig. 5:**
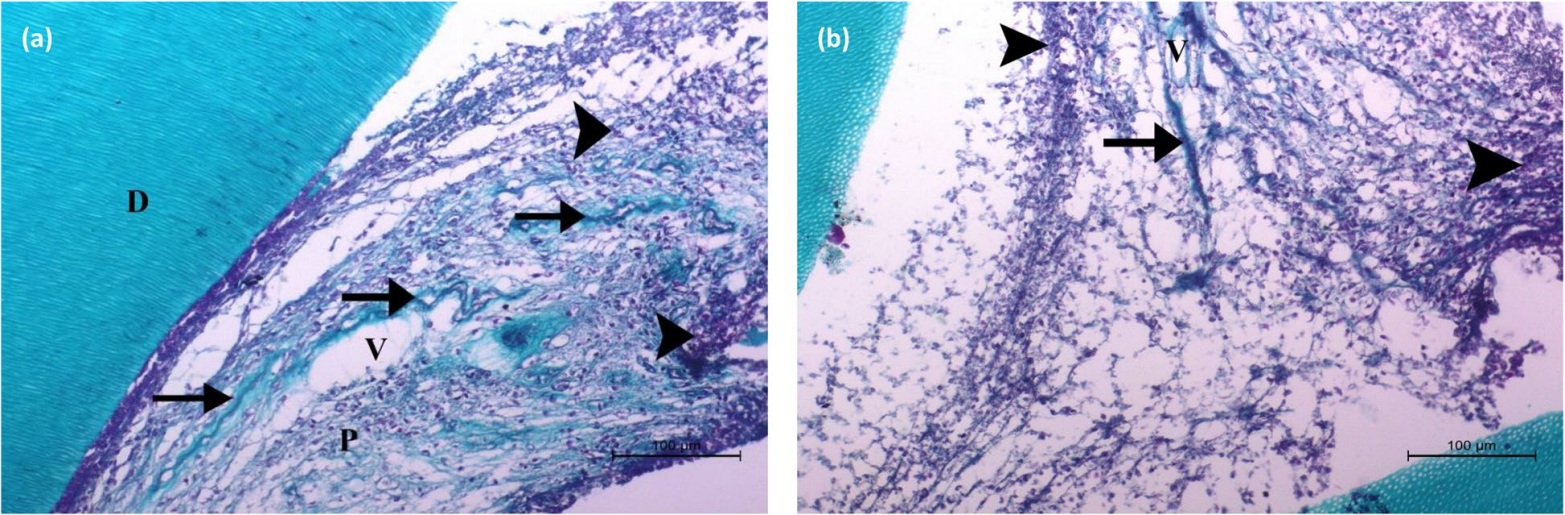
Photomicrograph of the dog's premolars capped with sodium hexametaphosphate (SHMP) (**a**) and mineral trioxide aggregate (MTA) (**b**), stained with Masson's trichrome. SHMP shows collagen bundles (black arrows) of the pulp (P) with few inflammatory cells (arrowheads) and normal blood vessels (V). MTA shows collagen bundles (black arrows) of the pulp (P) with many inflammatory cells (arrowheads) and congested blood vessels (V) (Scale bar 100 μm)

The photomicrograph of the pulp tissue of specimens stained with Masson’s trichrome showed that SHMP specimens had fewer chronic inflammatory cells and normal blood vessels compared to many inflammatory cells and congested blood vessels in the MTA specimens (Fig. 5).

### Findings of radiographic analysis

**Fig. 6:**
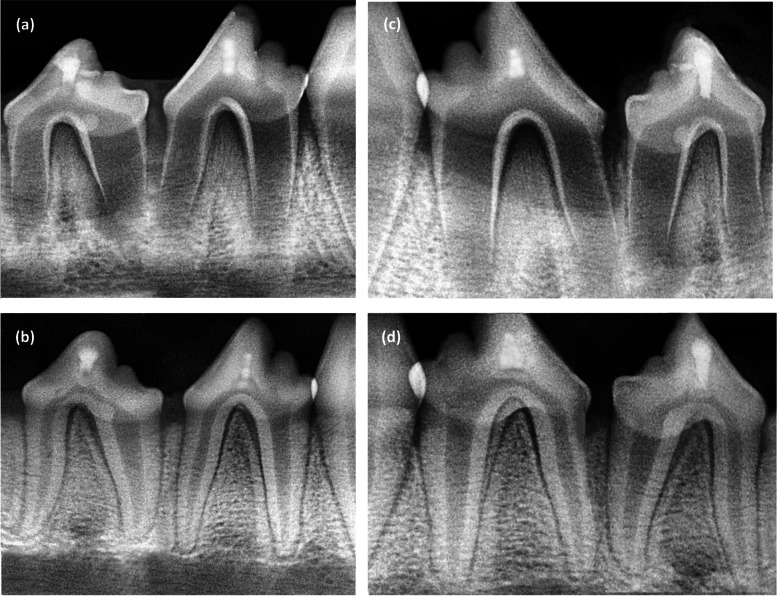
Periapical digital radiographs of dog's premolars show the change in mesial and distal root lengths (RL), apical foramen width (AFW), and root surface area (RSA) after capping with SHMP (**a** and **b**) and MTA (**c** and **d**). Radiographic parameter measures at baseline (**a**) and (**c**) and Radiographic parameter measures at 3 months (**b**) and (**d**)

After three months, the mean differencs in mesial and distal root lengths of premolars capped with SHMP and MTA were 0.42 mm with 95% CI of 0.13; 0.72 (*P* = 0.008) and 0.33 mm with 95% CI of 0.03; 0.64 (*P* = 0.048), respectively. Regarding the other radiographic parameters (RSA and AFW), both capping materials acted in a comprable manner with no statistically significant difference (*P* > 0.05) (Table 2) and (Fig. 6).

**Table 2:** Mean scores of different radiographic indicators of root maturation

Radiographic parameters	SHMP	MTA	95% *CI* of mean difference	P*
**Root length (RL/mm)**				
Mesial RL at baseline	4.18 ± 0.19	4.16 ± 0.10	-0.08; 0.13	0.66
Mesial RL after 3 months	6.53 ± 0.44	6.11 ± 0.44	**0.13; 0.72**	**0.008**
Distal RL at baseline	3.73 ± 0.25	3.86 ± 0.27	-0.35; 0.09	0.24
Distal RL after 3 months	6.46 ± 0.47	6.13 ± 0.46	**0.03; 0.64**	**0.048**
**Root surface area (RSA/mm**^**2**^**)**				
Mesial RSA at baseline	1.83 ± 0.27	2.00 ± 0.29	-0.37; 0.04	0.18
Mesial RSA after 3 months	3.39 ± 0.42	3.47 ± 0.38	-0.29; 0.05	0.17
Distal RSA at baseline	1.84 ± 0.25	1.96 ± 0.20	-0.34; 0.17	0.5
Distal RSA after 3 months	3.83 ± 0.27	3.92 ± 0.19	-0.23; 0.04	0.16
**Apical foramen width (AFW/mm)**				
Mesial AFW at baseline	1.74 ± 0.27	1.86 ± 0.21	-0.28; 0.05	0.16
Mesial AFW after 3 months	0.41 ± 0.13	0.47 ± 0.08	-0.14; 0.02	0.14
Distal AFW at baseline	1.71 ± 0.18	1.74 ± 0.11	-0.13; 0.08	0.58
Distal AFW after 3 months	0.47 ± 0.11	0.44 ± 0.10	-0.06; 0.09	0.65

*SHMP* Sodium hexametaphosphate, *MTA* Mineral trioxide aggregate

^*^Paired sample t test

## Discussion

The results of histological analysis showed the superiority of the calcific bridge properties of SHMP over the MTA in terms of complete calcific barrier formation, the qualitative properties of dentin formed within the bridge, and the quantitative measures including the thickness of predentin and odontoblastic layers (*H*_*0*_ was rejected). While the findings of other histological criteria, including the thickness of the dentin bridge and pulpal inflammation or tissue necrosis, in the experimental and control groups were comparable (*H*_*0*_ was not rejected).

The search for a novel capping material that provides a better pulp response is crucial before assessing the material's success in clinical situations. Additionally, to overcome the shortcomings of traditional MTA, alternative calcium silicate cements such as Biodentine, pre-mixed TotalFill BC RRM Putty, and pulp capping material (PCM) showed comparable high shear bond strength (SBS) values after immediate placement of the final restoration [29]. Xavier et al. [30] reported comparable SBS to Biodentine™ and NuSmile® NeoMTA with higher SBS values obtained after applying an extra layer of hydrophobic resin over the adhesive and placing the composite resin restoration 7 days after applying the calcium silicate cement (delayed restoration). However, cytotoxicity of freshly prepared (i.e., before setting) Biodentine and TheraCal has been encountered [31, 32].

One of the important aspects of pulp tissue response and the quality of dentin bridge is the preoperative pulp status [33]. According to Ricucci et al. [34] histological analysis of the coronal and radicular pulp tissues was normal for teeth that have been clinically diagnosed with reversible or irreversible pulpitis with necrotic foci next to the pulp horn. Mild to moderate grade of inflammation modulates the regenerative power of the pulp tissues, while severe and/or chronic inflammation has a determinant influence on the pulp [31]. Therefore, the radical shift towards treating teeth with irreversible pulpitis via the use of bioactive capping materials has become a focus of attention [31, 33]. Another aspect of DPC success is the ability of the capping material to protect the pulp in a healthy state and trigger mineralized barrier formation with superior quantitative and qualitative characteristics [5].

The dentin bridge induced by SHMP was better than that produced by MTA. The high ability of SHMP to elicit calcified bridge formation could be attributed to its ability to increase the activity of the ALP enzyme by upregulating the expression of non-specific ALP and endopolyphosphatase genes [21, 35]. The expression of other genes of bone matrix proteins, including osteonectin, OPN, and OCN, could be upregulated by Poly(P) [36]. These trigger the differentiation and proliferation of odontoblasts of HDPCs and the subsequent deposition of calcified niches [37]. Furthermore, the increased expression of ALP mediated by the long chain of SHMP prompts higher production of polymeric phosphate (PPi) and monomeric phosphate (Pi) in an optimal balance [35]. This permits the production of Pi at optimal levels, which induces calcified foci formation [21, 38].

Another possible mechanism of action of Poly(P) is triggering the expression of the matrix metalloproteinase (MMP)−3 gene in response to the injury of dental pulp and the subsequent release of inflammatory mediators [23]. The results of a study conducted in rats on odontoblast-like cells (iPS-OD) suggested that upregulated MMP-3 genes elicited a hierarchy of activation of odontoblastic markers, including dentin sialophosphoprotein (DSPP) and dentin matrix protein-1 (DMP-1). As a result of the serially upregulated cascade of odontoblast markers mediated by MMP-3, the proliferation and osteogenic differentiation of odontoblast-like cells have increased [23].

An integral part of pulp regeneration is the potential to organize a network of vascular capillaries (i.e., angiogenesis). SHMP, as a member of Poly(P), has the potential to elicit the migration and differentiation of HDPCs into endothelial cells through the evident elevation of the angiogenic markers, indicating an up-regulatory effect of Poly(P) on the angiogenic genes [39].

The MTA-induced calcified barrier was evident in all specimens with a variable degree of mineralization, indicating that the mineralization process of the matrix was heterogeneous. This agreed with the findings of a previous histological animal model study [3]. Interestingly, the current study findings showed that the degree of mineralization of dentin bridges formed in response to SHMP capping material was significantly superior to those organized by MTA. This observation might denote the faster rate of mineralization reaction of SHMP compared to MTA. The rate of calcification could be considered a determinant criterion for pulp capping materials [4]. This observation was confirmed by the presence of connective tissue in half of the MTA specimens. This was in line with the findings of a prior histological analysis [40] that connective tissue was detected in 40 percent of DPC samples. The results of the current study revealed that the newly formed odontoblast-like cell layer has a well-organized tubular system that could reflect the adequate surface adherence properties of the capping material. This prompts the better organization and differentiation of odontoblast-like cells [41].

Depending on the formation of the dentin bridge exclusively as an indicator of capping material success is not enough to justify a healthy pulp status [42]. There have been no previous reports regarding the pulpal inflammation response of SHMP. According to the findings of the present study, all specimens were free from inflammatory pulpal responses. The absence of pulp tissue inflammation and the highly qualitative and quantitative properties of the induced calcified tissue bridge refer to the superior bioinductive and biocompatible nature of the SHMP capping agent.

Regarding the MTA-induced calcified bridge, the aqueous medium provides a suitable environment for calcium hydroxide formation [4, 43]. At the interface between the material and pulp tissues, calcite-like structures are precipitated [44]. The developed crystals could attract the fibronectin required for cell adhesion and differentiation [4, 44]. The initial caustic pH level of MTA (pH of 12.5) at three hours following mixing [45]. The high pH continues up to eight weeks following material hardening [4, 46]. The high alkaline pH provides an optimum microenvironment for hard tissue barrier induction through the regulated release of cytokines and controlled inflammatory processes [4, 45].

Radiographic analysis of the teeth capped with both capping agents permits root maturogenesis in terms of the increase in RL and RSA and the closure of the apexes of dogs' premolars. However, the mesial and distal roots of teeth capped with SHMP revealed a significant increase in their RLs compared to those treated with MTA. Other radiographic parameters showed comparable results between the two materials. The relative radiographic superiority of SHMP over MTA emphasized the advantageous findings of histological analysis. The relative advantage in radiography could be attributed to the better healing conditions in terms of better qualitative and quantitative calcified bridge formation and less induced inflammation obtained by SHMP at the site of pulp injury. This favorable environment may encourage a faster healing rate compared to the MTA group. However, this point should be examined deeply in further investigations, as the histological and radiographic examinations are carried out over more than one period.

In the current study, a 3-month interval was adopted before considering histological and radiographic examination. This period was considered to ensure significant maturogenesis of the dog's roots. Moreover, this period provided a better chance for dentenogenesis to take place and permitted the maturation of the calcified dentin bridge. A similar interval was considered in a previous study [3], in which animals were sacrificed and specimens were assessed.

The main advantages of SHMP were the cost compared to the MTA. The 500 gm of SHMP costs 78.35 US$. Approximately each tooth was capped with 250 mg powder of SHMP or MTA. Thus, each tooth capped with SHMP costed about 3.92 cents, while the tooth capped with MTA costed about approximately 4.12 US$. The other merits of the use of SHMP include the ability to apply the final adhesive restoration with no obvious crown discoloration compared to crowns of teeth treated with MTA. However, further investigations are required to elucidate the long-term effect of SHMP on the color change and mechanical properties.

The main limitations of the present study are that it was performed in an ideal environment with sound dogs' teeth, healthy pulps with no previous inflammation, and standardized exposure sizes and sites. Therefore, it cannot guarantee that the response of the pulp tissues with preoperative inflammation will be similar [33]. The reaction of the pulp is totally different according to the type of exposure (traumatic injury, mechanical stimulus, or caries) [47]. Initially after the exposure, neuropeptides are released because of adjacent neural damage that is associated with an increase in vascular permeability [47]. The predominant inflammatory cells in the initial phase are lymphocytes, plasma cells, and macrophages associated with polymorphonuclear leukocytes in the acute phase, then the condition becomes chronic [48]. The chemotaxis of large numbers of neutrophils at the exposure site. This induces NETosis as a defensive mechanism which occurs because of the death of neutrophils. In NETosis, neutrophil extracellular traps destroy the invasive bacteria [49]. Mild to moderate inflammation is associated with reactive or reparative dentinogenesis, while severe inflammation may destroy the odontoblasts [50].

As previously mentioned, an early histological assessment might be required to provide a comprehensive perception regarding the inflammatory changes and the rate of healing of the SHMP capping material. Further studies are required to test the antimicrobial and sealing characteristics of SHMP and its sealing ability. Additionally, analysis of teeth with pulp exposure because of carious lesions is needed. Finally, assessment of the root maturogenesis depended on two-dimensional images. Therefore, future studies using 3D radiographs will be more beneficial. Additionally, further prospective clinical trials are required to confirm the effectiveness of SHMP and generalize the results of the present study.

## Conclusions

Within the limitations of the present study, it can be concluded that:

1. There was no difference in some respects between SHMP and MTA.
2. The histological evaluation showed that SHMP provided better bioinductive and biocompatible properties compared to MTA.
3. Radiographically, both materials showed comparable root maturogenesis outcomes except for the increase in RL, which was significantly longer after DPC with SHMP.
4. SHMP might be a suitable DPC alternative material in the treatment of immature permanent teeth.
5. Further prospective randomized clinical trials are necessary to prove the findings of the current study.

## Data Availability

Data sharing is not applicable to this article as no datasets were generated or analyzed during the current study. Access to data files and further information is available upon request by contacting Ahmad Elheeny, ahmedelheeny@mu.edu.eg.

## Abbreviations

SHMP: Sodium hexametaphosphate
DPC: Direct pulp capping
Poly(P): Inorganic polyphosphates
MTA: Mineral trioxide aggregate
VPT: Vital pulp therapy
